# Prevalence of Hepatitis E Virus Infection in Pigs at the Time of Slaughter, United Kingdom, 2013

**DOI:** 10.3201/eid2108.141995

**Published:** 2015-08

**Authors:** Sylvia Grierson, Judith Heaney, Tanya Cheney, Dilys Morgan, Stephen Wyllie, Laura Powell, Donald Smith, Samreen Ijaz, Falko Steinbach, Bhudipa Choudhury, Richard S. Tedder

**Affiliations:** Animal and Plant Health Agency, Addlestone, United Kingdom (S. Grierson, T. Cheney, S. Wyllie, L. Powell, F. Steinbach, B. Choudhury);; Public Health England, London, United Kingdom (J. Heaney, D. Morgan, S. Ijaz, R.S. Tedder);; University of Edinburgh, Edinburgh, Scotland, United Kingdom (D. Smith);; University of Surrey, Guildford, United Kingdom (F. Steinbach);; University College London, London (R. S. Tedder)

**Keywords:** Hepatitis E virus, seroprevalence, HEV RNA, genotype, phylogeny, pigs, public health, slaughter, viruses, United Kingdom

## Abstract

Since 2010, reports of infection with hepatitis E virus (HEV) have increased in England and Wales. Despite mounting evidence regarding the zoonotic potential of porcine HEV, there are limited data on its prevalence in pigs in the United Kingdom. We investigated antibody prevalence, active infection, and virus variation in serum and cecal content samples from 629 pigs at slaughter. Prevalence of antibodies to HEV was 92.8% (584/629), and HEV RNA was detected in 15% of cecal contents (93/629), 3% of plasma samples (22/629), and 2% of both (14/629). However, although HEV is prevalent in pigs in the United Kingdom and viremic pigs are entering the food chain, most (22/23) viral sequences clustered separately from the dominant type seen in humans. Thus, pigs raised in the United Kingdom are unlikely to be the main source of human HEV infections in the United Kingdom. Further research is needed to identify the source of these infections.

## Abstract 2

Pigs raised in the United Kingdom are unlikely to be the source of UK human infections.

Hepatitis E virus (HEV) that infects humans is composed of 4 genotypes (G1–4), each with a different geographic distribution and host range (*1*)*.* Although G1 and G2 infect humans only, G3 and G4 infect humans and animals. HEV G3 and G4 are distributed worldwide, with G3 most commonly infecting both humans and pigs in Europe (*2*)*.* From the observed incidence of acute HEV infection in blood donors (*3*), it is clear that HEV G3 infection in humans in England is far more common than previously thought. Realistic estimates are >100,000 infections annually.

Public Health England instituted enhanced surveillance of HEV infections in England and Wales in 2003 (*4*) and identified a recent and marked increase in the number of patients seeking treatment for HEV infections. In 2013, a total of 691 cases were identified, of which 477 (69%) were considered indigenous (occurring in persons who had not traveled outside England and Wales). Sequencing of strains from these acutely infected persons has identified an emergent phylogenetic cluster of HEV G3 infection in humans, which is likely to represent a zoonosis acquired through the consumption of undercooked meat (B. Said, pers. comm.).

In an early study in the United Kingdom of porcine samples archived during 1991–2001, antibodies to HEV were detected in 85.5% of 256 samples tested (*5*)*.* More recent studies across Europe indicate that many pig herds show evidence of HEV G3 infection (*6**–**10*)*.* A transient viremia in pigs is associated with dissemination of HEV into muscle and other tissues (*11*). A recent UK study found HEV RNA in 6 of 63 pork sausages tested, of which 5 were in a single batch of 11 (*12*), and a case−control study in England and Wales showed that human consumption of processed pork products is associated with an increased risk of acquiring HEV (*13*). It has long been considered plausible that the persistence of viremia in infected pigs up to the time of slaughter could provide a potential vehicle for zoonotic transmission to humans (*14*). We conducted surveillance of pigs at slaughter to investigate the epizoology of HEV in the United Kingdom and the extent of infection at the time pigs enter the food chain.

## Methods

### Study Details

Sample collection was undertaken during January–May 2013 as part of the 2013 Zoonoses in UK Pigs Abattoir Study, a cross-sectional study of pigs being slaughtered at 14 high-throughput abattoirs (12 in England and 2 in Northern Ireland) that together process 80% of all slaughtered pigs in the United Kingdom. The target population was all slaughtered pigs (finishers, cull sows, and boars) in the United Kingdom, excluding any condemned carcasses, pigs with a live weight <50 kg, pigs that had undergone emergency slaughter, and pigs that had been kept in the UK <3 months before slaughter (the latter was estimated to be around 2% of all slaughtered pigs) (*15*). Sampling was weighted so that the number of carcasses sampled in each of the selected abattoirs was proportional to the throughput of that abattoir and stratified by calendar month. Analysis was undertaken by using Stata statistical software version 12 (StataCorp LP, College Station, TX, USA). The prevalence estimates were calculated by using the svy command to adjust CIs because some pigs originated from the same farm.

### Samples

Whole blood taken by jugular vein stab and anticoagulated with EDTA was collected from 643 pigs for testing for antibodies to HEV and HEV RNA. Cecal contents from 638 pigs were available for testing for HEV RNA.

Paired plasma and cecal content samples were available for 629 pigs that met the study inclusion criteria. The pigs originated from 439 farms, with 1–10 pigs sampled per farm. The mode and median number of pigs per farm was 1. The geographic distribution of the pigs sampled was broadly proportionate to the UK pig population (*16*), and most (560, 89.0%) were <12 months old.

### Detection, Quantification, and Characterization of HEV RNA

Plasma HEV RNA was detected in nucleic acid extracts of plasma by using a quantitative TaqMan quantitative reverse transcription PCR assay (*12*) and expressed in international units per milliliter by comparison with the World Health Organization international standard. The limit of detection, defined by Poisson titration, was 22 IU/mL. Extracts whose amplification was below the threshold level of quantification of 100 IU/mL were confirmed to contain HEV RNA through amplification in a second PCR by using inner primers JVHEVF and JVHEVR (*17**,**18*)*.* Cecal content HEV RNA was detected in nucleic acid extracts of 10% fecal suspensions by using the TaqMan assay and a modified forward primer (JHEVF2, 5′-RGTGGTTTCTGGRGTGAC-3′), which gave a limit of detection of 250 IU/mL in cecal contents (25 IU/mL in 25% of replicates).

Phylogenetic analysis was attempted on all samples containing quantifiable HEV RNA detectable above a lower limit threshold corresponding to a cycle threshold (C_t_) value of 40 and on a proportion of lower samples. HEV open reading frame 2 (ORF2) (348-bp) fragments that could be amplified by nested PCR (*19*) were sequenced as previously described (*20*). Sequences were assembled into phylogenetic trees and compared with current UK human and porcine sequences retrieved from GenBank by using MEGA 6.0 (*21*)*.*

### Detection and Measurement of Antibodies against HEV

Antibodies against HEV were detected in swine plasma samples by using the Wantai Total HEV Antibody kit (Fortress Diagnostics Ltd., Antrim, UK) in accordance with the manufacturer’s protocol. To increase the dynamic range for approximate quantification of antibody, we retested samples with binding ratios (BR) >20 at a 1:10 dilution and adjusted the resulting BRs by a factor of 10.

Swine IgM against HEV was detected by using a modification of the human Wantai IgM assay in which the solid phase was replaced with microtiter wells coated with antibodies to the IgM-specific heavy chain domain of IgM (Bethyl Laboratories Inc., Montgomery, TX, USA). Reactivity was quantified by comparison with a calibration curve of a strong IgM-positive pig plasma arbitrarily attributed a potency of 100 pig IgM units/mL (100 AU/mL) serially diluted in HEV antibody-negative pooled pig plasma. Samples with reactivity >3.3 AU/mL in the presence of antibodies against HEV in other assays were considered to contain IgM. In the absence of HEV antibody reactivity, a more stringent cutoff of >10 AU/mL was used.

Swine IgG antibody was sought in an indirect immunoassay by using a modification of the Wantai IgG test for human serum samples where the labeled human IgG conjugate was replaced with labeled swine IgG (AbD Serotec, Kidlington, UK). A conservative cutoff optical density (OD) of 0.33 was used for this assay, determined by calculating the mean OD in the indirect assay of samples negative in the Wantai total assay, removing outliers, rederiving the mean negative OD, and using 1.5 × derived mean as cutoff. Samples giving reactions in excess of this were considered to contain HEV IgG.

## Results

Of 629 paired samples, 14 contained detectable HEV RNA in plasma and cecal samples. RNA was detected in 22 additional plasma samples and in 93 additional cecal samples. The prevalence of current infection defined by detectable plasma HEV RNA at any level, adjusting for clustering within farms, was 5.7% (95% CI 3.9%–7.6%). Similarly, the prevalence of current infection, defined by detection of HEV RNA in cecal samples at any level, again adjusting for clustering within farms, was 17.0% (95% CI 14.0%–20.0%). Taking detection of HEV RNA in either plasma or cecal samples as evidence of infection in 129 animals, we determined that the prevalence of current infection in pigs at slaughter was 20.5% (95% CI 17.2%–23.8%).

The viral load in the plasma ranged from detectable but below the limit of quantification to a maximum of 10^6^ IU/mL. Similarly, values in cecal content ranged from 40 to 7.4 × 10^7^ IU/mL. Six plasma samples contained in excess of 10^2^ IU/mL HEV RNA. Although high-level viremia in plasma samples was generally reflected by a high level of shedding in the cecal content (Table 1), most HEV RNA signals in either plasma or cecal content were present in only 1 of these paired sample types and then often only at low levels (plasma only mean C_t_ 40.5, range 34.3 to below levels of quantitation; cecal only mean C_t_ 37.2, range 24.2–45).

**Table 1:** Serologic analysis of and viral RNA in cecal and plasma samples in 6 pigs ranked by viremia whose HEV plasma load at slaughter exceeded 10^2^ IU/mL, United Kingdom*

Pig viral RNA				Serologic result†			
Cecal C_t_‡	Plasma C_t_	Viremia, IU/mL§	Wantai BR	IgG BR	IgM, AU/mL	Interpretation
24.23	23.7	5.95 × 10^5^		161	11.9	80.76	Current acute
24.71	25.5	1.92 × 10^5^		1.03	(0.7)	(2.91)	Early acute
33.04	34.7	4.40 × 10^3^		198	10.3	41.71	Current acute
37.46	34.2	2.80 × 10^3^		299	11.6	8.94	Late acute
Not detected	34.3	570		86	11.3	4.41	Late acute
39.34	37.3	270		85	7.5	4.73	Late acute

*AU, absorbance units; BR, binding ratio test/cutoff; C_t_, cycle threshold; HEV, hepatitis E virus. †Parentheses indicate BRs below cutoff. ‡C_t_ for amplification trace. §Interpolated IU/mL.

A total of 584 of 629 pig plasma samples were positive by manufacturers’ criteria in the Wantai total antibody assay (BR 1.0), and the pigs from which these samples were taken were considered seropositive. After adjusting for clustering of pigs within farms, we determined a seroprevalence of 92.8% (95% CI 90.7%–95.0%). Of the 584 plasma samples positive for antibodies against HEV, 276 (47.4%) also contained measurable IgM (reactivity >3.3 AU/mL), indicating a relatively recent infection. The 45 (7.2%) plasma samples from the survey that were unreactive by Wantai were tested for HEV IgM and IgG. Fifteen contained HEV IgG, albeit at low levels (mean BR 1.7, range 1.1–3.1), and 3 of the 15 contained HEV IgM (66.1, 33.4, and 18.3 AU/mL). Two of 30 that were unreactive for HEV IgG contained HEV IgM only (11.3 and 14.1 AU/mL).

Most (91%, 117/129) pigs with detectable HEV RNA at any site were seropositive, and half of the virus-positive pigs (48%, 56/117) also had detectable HEV IgM (IgM reactivity ≥3.3 AU/mL; Table 2). The remaining 220 seropositive pigs with detectable IgM did not have detectable HEV RNA. Pigs with high-level cecal content and plasma viremia had current or recent acute infection, and all had HEV IgM (Table 1). Seven of 9 seropositive pigs that were both viremic and shedding virus in the cecal content were seropositive for HEV IgM.

**Table 2:** Serologic status of 129 pigs in whom HEV RNA was detected in plasma, cecal fluid, or both, United Kingdom*

RNA-positive analyte	No. pigs	Pig serostatus†	
		No. positive (no. IgM positive)	No. negative
Plasma only	22	19 (8)	3
Plasma and cecal fluid	14	10 (8)	4
Cecal fluid only	93	88 (40)	5
Total	129	117 (56)	12

*HEV, hepatitis E virus. †Defined by Wantai test for antibodies against HEV.

Twelve infections were identified in pigs that were seronegative by manufacturers’ criteria in the Wantai assay in which HEV RNA was present at low level in cecal contents, plasma samples, or both (Table 2). Additional serologic testing of the 4 samples from Wantai seronegative pigs in which HEV RNA was detected in both cecal fluid and plasma (Table 3) showed 1 plasma sample to contain IgG just over the cutoff, another to contain low levels of IgM (3.4 AU/mL), and 2 samples to be reactive but below cutoff for IgG and IgM.

**Table 3:** Markers in the 4 Wantai antibody-seronegative plasma samples from pigs with concordant HEV RNA in plasma and cecal fluid samples, United Kingdom*

Cecal C_t_†	Viremia, IU/mL‡	Serologic results for HEV antibody		
		Wantai BR‡	IgG BR	IgM, AU/mL
37.49	BLQ	0.02	0.4	3.40
39.73	BLQ	0.02	0.7	2.20
39.62	69	0.22	0.4	1.50
39.66	45	0.35	1.2	0.80

*AU, absorbance units; BLQ, below the level of quantification; BR, binding ratio test/cutoff; C_t_, cycle threshold; HEV, hepatitis E virus. †C_t_ for amplification trace. ‡Interpolated IU/mL.

Amplification of RNA for sequencing was attempted on all samples containing quantifiable HEV RNA. However, due to low levels of input, virus sequences were only derived from 6 pig plasma and 21 cecal content samples. Where viral RNA could be sequenced from both plasma and cecal content, the animals had identical virus sequences at both sites (n = 4). All of the 23 unique sequences belonged to G3; all but 1 clustered within the UK human group 1 (Figure). A single cecal-derived sequence clustered within group 2 (*4*). Most viruses from UK patients with acute hepatitis clustered within group 2.

**Figure:**
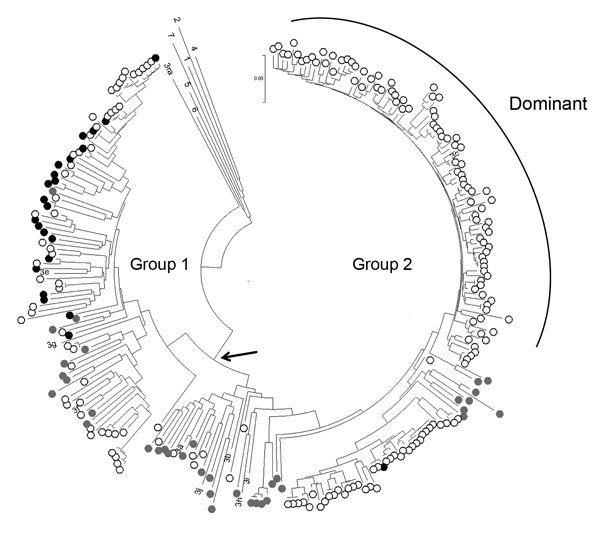
Phylogeny of genotype 3 hepatitis E viruses (HEVs) from pigs and patients with acute hepatitis in the United Kingdom. Nucleotide sequences of a 304-nt open reading frame 2 fragment (positions 5994–6297 of reference sequence M73218) from pigs at slaughter (black dots, n = 23) or from cases in persons with acute hepatitis E in England and Wales in 2013 (open circles, n = 190) were used to produce a neighbor-joining tree on the basis of maximum composite likelihood distances. GenBank accession numbers for porcine HEV sequences from this study are KP293752–774. Reference sequences were porcine sequences from Europe and North America from GenBank (gray dots, n = 36) and single examples of previously assigned HEV genotypes and subtypes for which complete genome sequences were available: 1, M73218; 2, M74506; 4, AJ272108; 5, AB573435; 6, AB602441; 7, KJ496143; 3a, AF082843; 3b, AB291955; 3c, FJ705359; 3e, AB248521; 3f, EU723514; 3g, AF455784; 3h, AB290312; 3i, FJ998008; 3j, AY115488; and 3ra, GU937805. Bootstrap support (500 replicates) for all major nodes, including those for genotypes 1–7, was weak (40%–70%), reflecting the short genome region and the large number of sequences analyzed. Arrow indicates the group 1/2 node.

## Discussion

We have demonstrated that HEV G3 RNA was present in plasma, cecal contents, or both in 20.5% of all pigs tested, which is likely to approximate the lower limit for a farm prevalence, given the modal number of pigs per farm sampled. A recent survey in France described a farm-level prevalence of 30% for HEV G3, with at least 1 RNA-positive animal detected in 27 of 90 farms sampled (*22*)*.* Similar findings in Canada (*23*) and Italy (*24*) have been documented for other tissues and organs. In Spain, 11.5% of liver or bile samples contained HEV RNA (*25*). 

Porcine HEV is acquired through ingestion of virus (*26*), and the extended duration of cecal fluid virus and, therefore, fecally shed virus in this study, exceeding that of the more transient plasma viremia (*11*), will favor porcine fecal-oral transmission. Extended fecal shedding means that older but immunologically naive animals joining the groups at finisher units may become infected closer to the time of slaughter. High cecal carriage and fecal excretion in some animals indicates the potential existence of superspreaders around the time of slaughter. The resulting environmental contamination may complicate attempts to control onward transmission of HEV in pigs.

High-level viremia was unusual, occurring in only 6 pigs, but although rare, this represents the best candidate source of potential dietary transmissions by meat ingestion (*27*)*.* Of these 6 pigs, 1 infection was in the early acute seroconversion phase. Two were in the acute phase of the infection, with high IgM levels, and the remaining 3 were later in the acute infection, with low IgM levels. All 6 pigs had detectable plasma IgM (Table 1), which probably indicates recent infections. We postulate that plasma viremia is a good marker for possible dietary transmission by meat products. The reported absence of porcine adenovirus (another virus found in pig feces) in HEV-contaminated sausages (*12*) also implicates viremia as the source of virus rather than fecal contamination at the abattoir.

We have reported (*4*) that the viruses causing current cases of G3 hepatitis E in humans fall into 2 phylogenetically and temporally separable groups, 1 and 2. These groups derive from the analysis of a 304-nt fragment of ORF2 with levels of bootstrap support in the region of 70% depending on the number of sequences analyzed. Much stronger support for these 2 groups is obtained when a larger 1,300-nt region of ORF2 is analyzed (data not shown). Most sequences of strains in humans contemporary to this study fall within group 2 (along with reference sequence 3c; Figure). In contrast, most G3 HEV (22 of 23) sequences obtained from UK pigs fall into group 1 (along with reference sequences of 3e, 3f, and 3g; Figure). Notably, the group 1 pig viruses are almost identical to those circulating in UK pig populations a decade ago (data not shown), perhaps demonstrating a longstanding zoonosis that may be reflected in the continuing group 1 cases in humans in England and Wales. The sole group 2 G3 HEV was from a pig from Scotland and falls outside the dominant human clade, sitting among a minor grouping. 

In England, as in most Western industrialized countries, HEV infection in humans comprises travel-associated (G1 and G3; potentially G2 and G4) and indigenous (G3) infections. Our findings indicates that, in the United Kingdom, indigenous HEV human-to-human infection will be rare, and nontravel-related hepatitis E results from HEV G3 dietary acquisition, as shown by recent and continuing case−control studies (*13*)*.*

Our findings suggest that slaughtered UK pigs are unlikely to be the source of most HEV G3 infections in humans in England and Wales. Although one could postulate the coexistence of group 2 viruses circulating in UK pigs, the failure to detect this virus at the time of slaughter in 22 of 23 pigs from whom virus could be sequenced would seem to render unlikely high-level viremia and possible infectivity of group 2 viruses through the contribution of UK pig meat to the food chain. We were not able to sequence most infections identified because of low viral levels in the reactive analytes. Consequent with our current understanding about infections in humans, it is instead plausible that the dominant HEV infections in humans that could be linked to pork consumption (*9*) derive from imported meat or meat products, although we are unable to establish the precise source. Other routes of transmission from hitherto unidentified animal sources to humans also cannot be excluded.

The GenBank sequences most closely related to the dominant clade of UK human HEV sequences derive from a wild boar from Germany sampled in 2006 or from pigs from Italy and France, all of which were sampled more than a decade ago. Trade of both pigs and pig products is a common practice in Europe, with imports accounting for around half of the pig meat consumed in the United Kingdom (*15*). Some of the acute hepatitis cases in UK patients associated with HEV G3 infection clustered together with most UK pig HEV isolates and the 3e reference sequence, and it is possible that UK pork is one of the sources of these infections. However, our findings conversely suggest that additional sources of pork may be responsible for further cases of human G3 HEV acute hepatitis clustering outside UK pig sequences.

The timing of HEV infection in pigs is a key consideration for informing future management options for mitigation of risk to public health. Because just over half of the pigs in this study had recently cleared infection, exhibiting antibodies against HEV and detectable IgM in the absence of detectable HEV RNA, they were likely to have been infected in the 2 months before slaughter. In this study, a few pigs remained seronegative at the time of slaughter (7.1%, or 4.5% if the 17 Wantai-negative samples reactive for IgG and IgM are considered seropositive) (Table 3). Our data indicate a lower prevalence of susceptibility (seronegativity) at slaughter in UK pigs than in continental Europe (*7**–**10**,**21**,**28*).

The demonstration of pigs viremic at time of slaughter explains the detection of HEV in processed food products in the absence of apparent hygiene problems. It seems likely that we are measuring by proxy, through the infection of humans, the spread of group 2 HEV G3 infection in pig populations that supply meat to the United Kingdom. The continuing annual increase in hepatitis E cases in humans in the United Kingdom may reflect changes in trade, processing, or husbandry in other countries, or a societal change in how pig meat is consumed. Consideration has to be given to developing a better understanding of this widespread zoonosis. Given the ubiquity and transmissibility of porcine HEV infection in swine, with the simultaneous absence of clinical signs, economic effects, or regulation, the elimination of HEV in pigs is unlikely in the near future.

Immunization of pigs against HEV is currently only a theoretical option because there no vaccines are on the market for pigs. In addition, one would have to ensure that any intervention is not merely delaying infection and increasing the likelihood of viremia at the time of slaughter (*29**,**30*). An alternative approach through using husbandry practices to facilitate natural immunity in early life should also be considered. Further investigations into HEV infection in humans and pigs in the United Kingdom and other countries are required to inform farming management practices to reduce active porcine infection rates at the time of slaughter.
